# Personality Pathology and Cognitive Aging

**DOI:** 10.1093/geroni/igaa057.1252

**Published:** 2020-12-16

**Authors:** Patrick Cruitt, Patrick Hill, Thomas Oltmanns

**Affiliations:** 1 Washington University in St. Louis, Saint Louis, Missouri, United States; 2 Washington University in St. Louis, St. Louis, Missouri, United States

## Abstract

Research on the relationship between normal-range personality and cognitive aging has demonstrated consistent, but modest, effects. The current investigation seeks to increase our understanding of unhealthy cognitive aging by examining the maladaptive extremes of personality. Borderline and avoidant personality disorder (PD), but not obsessive-compulsive PD, were hypothesized to show prospective associations with cognitive aging. The current investigation tested this hypothesis in two longitudinal studies of older adulthood: the Alzheimer’s Disease Research Center cohort (ADRC, N = 434, Mage = 69.95, 56% women) and the St. Louis Personality and Aging Network study (SPAN, N = 1,058, Mage = 65.92, 54% women). The ADRC study administered a battery of neuropsychological tests to assess cognitive ability/memory. Borderline PD was measured with a composite derived from the NEO Five Factor Inventory. The SPAN study administered self-, informant, and interview measures of the three PDs, a free recall memory task, and an informant report measure of cognitive problems. Borderline PD features exhibited cross-sectional correlations with memory (ADRC: r = -.11; SPAN: all rs = -.08), general cognitive ability (ADRC: r = -.11), and informant reported cognitive problems (rs ranged from .15 to .39). These features also prospectively predicted changes in cognitive problems (Std. bs = .13 and .15), but not in memory or cognitive ability. Avoidant and obsessive-compulsive PD exhibited little association with cognitive aging. These findings suggest that borderline PD features may interfere with cognitive maintenance interventions. Furthermore, they argue for the development of PD treatments adapted for the context of later life.

